# Epileptogenic high-frequency oscillations present larger amplitude both in mesial temporal and neocortical regions

**DOI:** 10.3389/fnhum.2022.984306

**Published:** 2022-09-29

**Authors:** Victor Karpychev, Alexandra Balatskaya, Nikita Utyashev, Nikita Pedyash, Andrey Zuev, Olga Dragoy, Tommaso Fedele

**Affiliations:** ^1^Center for Language and Brain, HSE University, Moscow, Russia; ^2^National Medical and Surgical Center named after N.I. Pirogov, Moscow, Russia; ^3^Institute of Linguistics, Russian Academy of Sciences, Moscow, Russia; ^4^Institute for Cognitive Neuroscience, HSE University, Moscow, Russia

**Keywords:** epilepsy surgery, intracranial EEG, high-frequency oscillations, seizure outcome, machine learning

## Abstract

High-frequency oscillations (HFO) are a promising biomarker for the identification of epileptogenic tissue. While HFO rates have been shown to predict seizure outcome, it is not yet clear whether their morphological features might improve this prediction. We validated HFO rates against seizure outcome and delineated the distribution of HFO morphological features. We collected stereo-EEG recordings from 20 patients (231 electrodes; 1,943 contacts). We computed HFO rates (the co-occurrence of ripples and fast ripples) through a validated automated detector during non-rapid eye movement sleep. Applying machine learning, we delineated HFO morphological features within and outside epileptogenic tissue across mesial temporal lobe (MTL) and Neocortex. HFO rates predicted seizure outcome with 85% accuracy, 79% specificity, 100% sensitivity, 100% negative predictive value, and 67% positive predictive value. The analysis of HFO features showed larger amplitude in the epileptogenic tissue, similar morphology for epileptogenic HFO in MTL and Neocortex, and larger amplitude for physiological HFO in MTL. We confirmed HFO rates as a reliable biomarker for epilepsy surgery and characterized the potential clinical relevance of HFO morphological features. Our results support the prospective use of HFO in epilepsy surgery and contribute to the anatomical mapping of HFO morphology.

## Introduction

High-frequency oscillations (HFO), also known as ripples (80–250 Hz) and fast ripples (FR, 250–500 Hz) have been associated with the epileptogenic area, required for the generation of epileptic seizures (Bragin et al., 1999; Rosenow and Lüders, 2001; Jiruska and Bragin, 2011), as well as the seizure onset zone, the area where the seizure is firstly observed (SOZ, Jirsch et al., 2006; Crépon et al., 2010; Jacobs et al., 2012). Retrospective group studies validated that the resection of tissue with high rates of FR and, to a lesser extent, ripples correlated with good seizure outcome (Jacobs et al., 2010; Cho et al., 2014; Kerber et al., 2014; Okanishi et al., 2014; van Klink et al., 2014).

However, prospective studies targeting the prediction in individual patients reported mixed results. While HFO could correctly predict seizure outcome in intraoperative (van’t Klooster et al., 2015) and pre-operative recordings (Fedele et al., 2017; Dimakopoulos et al., 2021), a multicenter study showed low predictive power of HFO with limited improvement in standard surgical planning (Jacobs et al., 2018). Moreover, the analysis of a large patients’ cohort highlighted high variability of HFO spatial distribution even during the same night (Gliske et al., 2018). Therefore, the suitability of HFO for clinical application is still under debate.

These conflicting results might be partially addressed to methodological differences in the HFO detection pipeline (Fedele et al., 2019). First, the data selection should follow the same criteria across studies. To date, non-rapid eye movement (NREM) sleep data provided higher rates for both ripples and FR than REM sleep or wakefulness (Staba et al., 2004; Bagshaw et al., 2009; Sakuraba et al., 2016; Klimes et al., 2019; Cserpan et al., 2021) and longer periods of NREM sleep significantly improved the prognostic value of FR rates (Nevalainen et al., 2020). Second, it is necessary to apply automated detectors of HFO prospectively validated against seizure outcome on different datasets (Zelmann et al., 2012; Fedele et al., 2017; Boran et al., 2019a,b, 2021; Dimakopoulos et al., 2021). Following these recommendations, we applied a successfully validated detector (Fedele et al., 2017, HFO as the co-occurrence of ripple and FR) on NREM sleep recordings in a newly collected dataset, characterized by extensive coverage of mesial temporal lobe (MTL) and Neocortex.

While HFO rates are the main information considered in the delineation of the epileptogenic tissue (Chen et al., 2021), ripples and FR are also present in the non-epileptogenic tissue (Engel et al., 2009; Frauscher et al., 2018). However, it is still unclear how to correctly classify epileptogenic HFO (Nagasawa et al., 2012; Frauscher et al., 2018). One possible approach is to examine HFO morphological features, such as amplitude, duration, and spectral frequency (Chen et al., 2021). When comparing HFO events detected in the epileptogenic vs. non-epileptogenic tissues, ripples showed larger amplitude, longer duration and lower frequency (von Ellenrieder et al., 2016, Charupanit et al., 2020), FR consistently showed larger amplitude but either shorter (Pail et al., 2017) or longer duration (von Ellenrieder et al., 2016) in the MTL, while neocortical HFO detected in the full spectral range showed either larger (Guragain et al., 2018) or lower amplitude (Alkawadri et al., 2014). In this study, rather than focusing on the statistical evaluation of the individual HFO morphological feature, we considered their multivariate information. Therefore, we applied a machine learning approach targeting single event classification (Sciaraffa et al., 2020; Segato et al., 2020) across all available electrode contacts and, separately, in MTL and Neocortex (Weiss et al., 2019).

Overall, this study aims to prospectively validate the HFO rates (the co-occurrence of ripple and FR) as a predictor of seizure outcome in a newly collected dataset, following specific recommendations for HFO detection. Moreover, we report on the retrospective delineation of the distribution of HFO morphological features in epileptogenic and non-epileptogenic tissues across all contacts and, separately, in MTL and Neocortex.

## Materials and methods

### Cohort

We prospectively considered consecutive patients with drug-resistant epilepsy who underwent invasive EEG recordings and resective or thermocoagulation surgery at National Medical and Surgical Center named after N.I. Pirogov (Moscow, Russia) between January 2017 and June 2021, with a follow-up period of at least 1 year. Seizure outcomes were classified according to the International League Against Epilepsy (ILAE). The surgical plan was independent from HFO analysis. We applied the following exclusion criteria: absence of NREM sleep, vagus nerve stimulation and corpus callosotomy as seizure treatment. All patients gave written informed consent. The study was approved by National Medical and Surgical Center named after N.I. Pirogov Ethics Committee.

### Recording techniques

For each patient, at least seven depth stereo-EEG electrodes (electrodes: mean = 11.6, SD = 3.3, range = 7–19; contacts: mean = 97.8, SD = 22.9, range = 53.0–128.0) were implanted to delineate the SOZ. We used post-implantation T1 or T2 MRI to anatomically localize each electrode contact through a semi-automated procedure (Stolk et al., 2018). Our anatomical mapping was confirmed by the surgeon (N.P.). Stereo-EEG data were acquired at a sampling frequency of either 2000 or 2048 Hz on multiple acquisition systems: Natus (Natus Medical Incorporated, CA, USA), EBNeuro (Galileo, Firenze, Italy), and MicroMed (MicroMed, Veneto, Italy). The three acquisition systems featured comparable noise level in the high frequency domain (Supplementary Figure 1). To identify intervals of NREM sleep for HFO detection, for each electrode, we considered the EEG signal from the contact closer to the scalp located in the neocortex (Reed et al., 2017). For all patients, we identified from four to 14 intervals of 5-min of NREM sleep. After excluding noisy contacts (mean = 16.5, SD = 10.0), the mean number of contacts per patient was 81.7 (SD = 24.2). We derived the bipolar montage along consecutive contacts for further analysis.

### Detection of ripples and fast ripples

To detect ripples and FR, we applied an automated detector (Fedele et al., 2017), which was clinically validated in previous studies (Fedele et al., 2017; Boran et al., 2019a,b, 2021; Cserpan et al., 2021; Dimakopoulos et al., 2021). The automated detector consists of two stages. In the first stage, events of interest exceeding an amplitude threshold defining the background activity were identified in the frequency range of ripples and FR. The amplitude threshold was computed from the cumulative distribution of the amplitude envelope of time segments exhibiting high Stockwell entropy (low probability of sustained oscillations). In the second stage, events of interest were tested against morphologic criteria in both ripple and FR ranges. Ripples with peak amplitude exceeding 30 μV and FR with peak amplitude exceeding 20 μV were rejected as artifacts. Similarly, a duration threshold of 150 ms was used for ripples; 50 ms was used for FR. We verified that our HFO detector provides events minimally overlapping with epileptic spikes. Details of the overlap between spikes and HFO events are reported in the Supplementary material (Supplementary Table 1).

### Test-retest reliability of high-frequency oscillations rate

In line with Fedele et al. (2017), we estimated the test-retest reliability of HFO rate over several 5-min NREM intervals within each. For each interval, we obtained the HFO rate spatial distribution as a vector, whose dimension is the number of bipolar channels. For all interval pairs, we obtained a distribution of the normalized scalar product across HFO rate. To test the magnitude of the true scalar product against chance, we constructed a distribution of scalar products by randomly permuting (*N* = 5000) the order of channels for each interval. The true value of the scalar product was considered statistically significant if it exceeded the 97.5 percentile of the distribution. We calculated the mean value of a scalar product expressing the test-retest reliability across all patients.

### Validation of high-frequency oscillations rate against seizure outcomes

For each patient, we obtained a spatial distribution of HFO rates across all available bipolar channels. We identified the HFO area as the ensemble of channels with at least one event per minute and exceeding the 95%-threshold of the HFO rate distribution (for different choices of the threshold percentile see Supplementary Figure 2). To evaluate the predictive power of the HFO rates, we considered a clinical case presenting seizure recurrence as positive (if seizure free as negative), and a correct prediction as true (an incorrect prediction as false). Thus, in line with previous studies (van’t Klooster et al., 2015; Fedele et al., 2017; Boran et al., 2019a,b; Dimakopoulos et al., 2021), we defined four categories of patients to quantify the predictive power of HFO rates against seizure outcome: true positive (TP) indicated non-resected or partly resected HFO areas in patients with poor outcome (ILAE = 2–6); false positive (FP) – non-resected or partly resected HFO areas in patients with good outcome (ILAE = 1); true negative (TN) – fully resected HFO areas in patients with good outcome; false negative (FN) – fully resected HFO areas in patients with poor outcome. We calculated the positive predictive value (PPV) = TP/(TP + FP); negative predictive value (NPV) = TN/(TN + FN); sensitivity = TP/(TP + FN); specificity = TN/(TN + FP); accuracy = (TP + TN)/N, with N being the number of patients.

### Extraction of high-frequency oscillations morphological features

To characterize HFO morphological features, we extracted the amplitude as the peak-to-peak voltage in ripples (Am-ripples) and FR (Am-FR), the central frequency as the inverse of the averaged interpeak distance in ripples (Fr-ripples) and FR (Fr-FR), and the duration as the difference between onset and offset of the HFO event (D-HFO).

### Evaluation of high-frequency oscillations morphological features

We applied *Random forest (RF)* classifiers to quantify the differences in HFO features between the epileptogenic and non-epileptogenic tissues across all contacts and, separately, in MTL and Neocortex. Following Guragain et al. (2018), we considered the hippocampus, parahippocampal gyrus, and amygdala as MTL, whereas the lateral temporal lobe and frontal, parietal, and occipital lobes as Neocortex. Similarly, we applied RF classifiers to quantify the differences in HFO features between MTL and Neocortex, separately, inside the epileptogenic and non-epileptogenic tissues. A RF classifier is an ensemble of decision trees, each of which trains on an individual bootstrap sample of the data using randomly chosen features at each decision node of a tree (Breiman, 2001; Liaw and Wiener, 2002). We built all RF classifiers using the package “*scikit-learn*” in Python 3.9 (Pedregosa et al., 2011). HFO features data and code for the RF classifiers are available online.^1^

#### Dataset of high-frequency oscillations morphological features

We divided HFO events into two classes. In Class-1 we included the HFO events detected in resected HFO areas in TN patients; in Class-2 we included the HFO events detected in non-resected areas in TN patients. Therefore, Class-1 corresponded to the epileptogenic tissue and Class-2 to the non-epileptogenic tissue, consistently with the surgical outcome. In each class of each patient, we removed outliers through an *Isolation Forest* approach (Liu et al., 2008). Every HFO feature was *z*-scored with mean and standard deviation computed across all events available in the full set of TN patients. We applied Mood’s median test to verify whether HFO features were separable between the classes considering electrode contacts across all contacts, in MTL and Neocortex (von Ellenrieder et al., 2016). Similarly, we applied Mood’s median test to verify whether HFO features were different between MTL and Neocortex, separately, inside each of both classes. Due to multiple testing for all classes, we adjusted the level of significance for 25 Mood’s median tests (α = 0.05/25 = 0.002). We did not consider the HFO events in TP, FP, and FN patients because we cannot be conclusive about their epileptogenicity. Thus, we retrospectively characterized differences in HFO morphological features of TN patients between the epileptogenic and non-epileptogenic tissues across all contacts, MTL and Neocortex.

#### Cross-validation procedure

We built our RF classifiers on the population of available events without distinguishing across different patients. Following previous studies (Jrad et al., 2017; Sciaraffa et al., 2020), we evaluated all RF classifiers through *nested cross-validation (CV)*, which consisted of fivefold inner and outer CV loops (Krstajic et al., 2014). This approach allows us to randomly divide the dataset into folds with the same number of events in both classes across all iterations. For comparison, we also applied *leave-one-patient-out* CV (see Supplementary Tables 4, 5).

For each RF classifier, within each fold of the inner CV loop, we applied *a Grid search* algorithm to find the optimal number of decision trees and nodes in each tree, bootstrap samples, and randomly chosen features at each node. These values further were used to train a RF classifier within each fold of the outer CV loop. We balanced the number of HFO events during training by the *Synthetic Minority Over-sampling Technique (SMOTE)* approach (Batista et al., 2004) using the package “*imbalanced-learn*” (Lemaître et al., 2017). We quantified the performance of each RF classifier computing the area under the receiver operating characteristic curve (AUC) and the importance of each feature using the mean decrease in the *Gini index* (Nembrini et al., 2018).

## Results

### Cohort

The patient cohort included 20 patients (10 females; age: mean = 34.4, SD = 10.2, range = 19–69 years; follow-up period: mean = 29.3, SD = 11.2, range = 13–45 months). Fourteen patients had temporal lobe epilepsy, 12 with mesial and two patients with lateral temporal lobe epilepsy. Among six patients with extratemporal lobe epilepsy, two had SOZ in the temporo-parietal area, two in the frontal lobe, one in the parietal lobe, and one in the insular lobe. Seizure freedom was achieved in 14 patients (ILAE = 1). Table 1 summarizes the clinical characteristics of the patient cohort.

**TABLE 1:** Patients’ clinical characteristics.

ID	Age, gender	Pre-operative MRI/ Pathology	Epilepsy, SOZ	Surgery, RA	Intervals, min	Test-retest reliability [*SD*],%	HFO area [Res/NRes]	Outcome (ILAE)	Status HFO rate	Follow-up period, months
1	28, F	MRI-negative FCD-IIb; L–FL	ETLE, L–FL	FRS, L–FL	25	82.6 [9.1]	L–HC, FL [NRes]	3	TP	45
2	36, F	CA; R–PL FCD-IIIc; R–TL	MTLE, R–HC	FRS, R–HC, TL	50	94.8 [2.4]	L–HC, R–HC [NRes]	4	TP	44
3	26, F	HS; R	MTLE, R–HC	FRS, R–HC, TL	45	81.7 [14.6]	R–HC, TL [Res]	1	TN	44
4	44, F	MRI-negative FCD-IIa; L–TL	MTLE, L–HC	FRS, L–HC, TL	30	90.1 [6.4]	L–HC [Res]	1	TN	43
5	33, F	HS; R	MTLE, R–HC	FRS, R–HC, TL	20	96.8 [1.7]	R–HC [Res]	1	TN	40
6	27, M	MRI-negative FCD-IIa; R–TL	ETLE, R–Insula	FRS, R–Insula, TL	60	74.5 [15.6]	R–HC, TL [Nres]	3	TP	40
7	30, F	Gliosis; L–TL FCD-IIIb; L–TL	ETLE, L–TL, PL	FRS, L–TL, PL	70	95.9 [2.2]	L–HC [Nres]	5	TP	38
8	30, M	HS; L	MTLE, L–HC	FRS, L–HC, TL	70	94.4 [6.5]	L–HC, PL [Nres]	1	FP	33
9	35, M	FCD-IIa; R–TL	MTLE, R–HC	FRS, R–HC, TL	50	68.2 [21.7]	R–HC [Res]	1	TN	31
10	44, M	MRI-negative HS; L	MTLE, L–H, Am	FRS, L–HC, Am, TL	40	81.1 [12.3]	L–HC, Am [Res]	1	TN	30
11	32, F	PNH; R–OL	MTLE, R–HC	FRS, R–HC, TL	35	96.4 [2.6]	R–HC, OL [NRes]	1	FP	30
12	37, M	MEC; R–TL	ETLE, R–PL	FRS, R–PL, OL	35	68.5 [10.3]	L–HC [NRes]	5	TP	28
13	39, M	Gliosis; R–TL, OL, PL FCD-IIIa; R–TL	MTLE, R–HC	FRS, R–HC, TL	30	73.8 [13.5]	R–HC [Res]	1	TN	23
14	39, M	MEC; L–TL	LTLE, R–TL	FRS, R–HC, TL	30	81.0 [14.8]	R–HC [Res]	1	TN	18
15	32, M	Gliosis; R–TL	LTLE, R–HC, TL	RT, R–HC, TL	30	92.9 [3.0]	R–HC, TL [Res]	1	TN	20
16	69, F	HS; L, CA; L–FL	MTLE, L–HC	RT, L–HC, TL	55	95.4 [3.6]	R–HC [Res]	1	TN	19
17	19, F	DA; L–MTL	ETLE, L–TL	RT, L–HC, TL	95	91.9 [8.0]	L–HC, PL [NRes]	3	TP	18
18	27, M	MRI-negative Gliosis; R–TL	MTLE, R–HC	FRS, R–HC, TL	45	82.9 [6.9]	L–HC, R–HC [NRes]	1	FP	16
19	28, M	MRI-negative HS; L	MTLE, L–HC	RT, L–HC, TL	30	97.7 [1.1]	L–HC [Res]	1	TN	13
20	32, F	FCD-II; R–FL	ETLE, R–FL, PL	RT, R–FL, PL	40	88.1 [6.8]	R–PL [Res]	1	TN	13

Am, amygdala; CA, cavernous angiomas; DA, developmental abnormalities; ETLE, extratemporal lobe epilepsy; FCD, focal cortical dysplasia; FL, frontal lobe; FRS, focal resection surgery; HC, hippocampus; HS, hippocampal sclerosis; ILAE, International League Against Epilepsy; LTLE, lateral temporal lobe epilepsy; L, left; MEC, meningoencephalocele; MTLE, mesial temporal lobe epilepsy; OL, occipital lobe; PL, parietal lobe; PNH, periventricular nodular heterotopia; R, right; RA, resected area; Res, resected; NRes, non-resected; RT, radiofrequency thermocoagulation; SD, standard deviation; SOZ, seizure onset zone; TL, temporal lobe.

### High-frequency oscillations detection and test-retest reliability of high-frequency oscillations

We applied HFO detection to the patient cohort during NREM sleep and assessed the stability of the HFO spatial profile across time. The weighted mean test-retest reliability across all patients was 86.9% (SD = 9.3%, range = 65.8–97.7%) over the NREM sleep intervals (duration: mean = 44.3 min, SD = 18.5 min, range = 20–95 min). Thus, the spatial profile of the HFO rate was stable in our study. In Table 1, we report the level of test-retest reliability for all patients.

### High-frequency oscillations rate predicted seizure outcome

**FIGURE 1:**
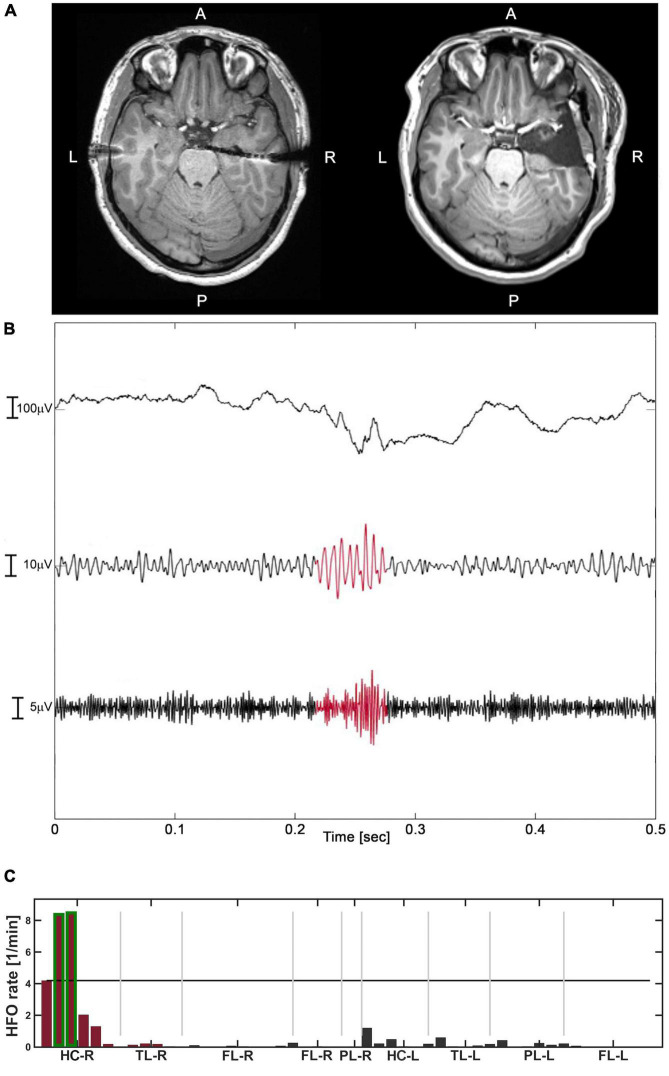
The analysis of Patient-3 (good outcome – TN). **(A)** Pre-operative T1 MRI (left) and post-operative T1 MRI (right). **(B)** An example of HFO, defined as the co-occurrence of ripple and FR, highlighted in red in their respective filtered range. **(C)** The distribution of HFO rates across channels (events/minute). The channels with HFO rate exceeding the 95%-threshold (black horizontal line) formed the HFO area, highlighted in green. The channels in the resected area are marked in red. The HFO area was included in the resection which led to good outcome (TN).

We describe our analysis workflow for the delineation of the HFO area in Figure 1 in one representative patient. Figure 1C shows the HFO rate spatial distribution, with resected channels marked in red and channels included in the HFO area highlighted in green. In this case, the HFO area was included in the resected area, which correctly predicted seizure freedom. Therefore, we classified this patient as TN. Additionally, we provided examples of HFO rate prediction against seizure outcome specifying stereo-EEG implantation scheme and signals at seizure onset in the Supplementary material for TN (Supplementary Figure 3) and TP (Supplementary Figure 4) patients. In our patient cohort, we had 11 TN, 6 TP, 3 FP, and no FN. Thus, HFO rate predicted seizure outcome with sensitivity and NPV of 100%, specificity of 79%, PPV of 67%, and accuracy of 85% (Table 2). Further discussion on the FP patients is provided in section “High accuracy of high-frequency oscillations rates for outcome prediction.”

**TABLE 2:** HFO prediction of seizure outcome.

	Definition	HFO rate
Specificity [*CI*], %	TN/(TN + FP)	79 [49 95]
Sensitivity [*CI*], %	TP/(TP + FN)	100 [54 100]
Negative Predictive Value (NPV) [*CI*], %	TN/(TN + FN)	100 [72 100]
Positive Predictive Value (PPV) [*CI*], %	TP/(TP + FP)	67 [30 93]
Accuracy [*CI*], %	(TN + TP)/N	85 [62 97]

CI, confidence interval; FN, false negative; FP, false positive; HFO rate, rates of the co-occurrence of ripple and FR; N, number of patients; TN, true negative; TP, true positive.

### Evaluation of high-frequency oscillations morphological features

Given the high accuracy of HFO analysis in this dataset, we aim to further characterize the morphology of epileptogenic HFO. We considered as relevant morphological features the amplitude and frequency in both ripple and FR ranges and the duration of HFO events (section “Extraction of high-frequency oscillations morphological features”). Next, we compared HFO morphological features in the epileptogenic and non-epileptogenic tissues across all contacts and, separately, in MTL and Neocortex. We considered TN patients, where the resection of HFO area is predictive of seizure freedom (section “Dataset of high-frequency oscillations morphological features”). Epileptogenic HFO (Class-1) included 5,507 events, 4,809 events in MTL, and 698 events in the Neocortex; physiological HFO (Class-2) included 1,929 events, 1,006 events in MTL, and 923 events in the Neocortex. Table 3 describes the comparison between Class-1 and Class-2 for each HFO feature across all contacts, in MTL and Neocortex, while Table 4 describes the comparison between MTL and Neocortex within Class-1 and Class-2.

**TABLE 3:** Results of Mood’s median test comparing HFO features between Class-1 and Class-2 across all contacts, in MTL and Neocortex.

	All contacts			MTL			Neocortex		
	Class-1	Class-2	χ ^2^_(1,7436)_	Class-1	Class-2	χ ^2^_(1,5815)_	Class-1	Class-2	χ ^2^_(1,1621)_
	Mdn [IQR]	Mdn [IQR]		Mdn [IQR]	Mdn [IQR]		Mdn [IQR]	Mdn [IQR]	
Am-FR	15.2 [9.9]	8.5 [4.9]	1233.6*	15.3 [9.7]	9.6 [4.7]	575.3*	14.3 [11.6]	7.1 [4.7]	419.7*
Am-ripples	37.8 [22.9]	27.6 [25.1]	241.2*	37.8 [22.6]	33.8 [23.6]	26.3*	37.8 [25.4]	19.4 [20.0]	233.2*
Fr-FR	375.5 [20.6]	376.8 [18.6]	9.3	375.7 [20.5]	377.6 [17.3]	12.1*	373.7 [20.9]	375.7 [19.5]	3.7
Fr-ripples	167.6 [19.9]	168.1 [18.2]	1.2	167.6 [19.4	167.6 [18.0]	0	167.9 [22.7]	169.1 [19.0]	0.6
D-HFO	70.5 [39.6]	75.0 [50.1]	18.9*	71.3 [39.6]	80.1 [50.8]	39.6*	63.7 [38.9]	68.5 [46.9]	6.39

Am-FR, amplitude of FR; Am-ripples, amplitude of ripples; D-HFO, duration of the co-occurrence of ripple and FR; Fr-FR, frequency of FR; Fr-ripples, frequency of ripples; IQR, interquartile range; Mdn, median; MTL, mesial temporal lobe.

*Difference significant at α = 0.002 Bonferroni corrected.

**TABLE 4:** Results of Mood’s median test comparing HFO features between MTL and Neocortex inside Class-1 and Class-2.

	Class-1		Class-2	
	χ ^2^_(1,5507)_	*P*-value	χ ^2^_(1,1929)_	*P*-value
Am-FR	7.1	0.008	200.3	< 0.001*
Am-ripples	0	1.0	177.8	< 0.001*
Fr-FR	4.8	0.03	6.4	0.01
Fr-ripples	0.1	0.70	2.6	0.11
D-HFO	20.3	< 0.001*	27.2	< 0.001*

Am-FR, amplitude of FR; Am-ripples, amplitude of ripples; D-HFO, duration of the co-occurrence of ripple and FR; Fr-FR, frequency of FR; Fr-ripples, frequency of ripples.

*Difference significant at α = 0.002 Bonferroni corrected.

For all contacts, MTL and Neocortex, we observed larger amplitude of both FR and ripples for HFO events detected in the epileptogenic tissue and, as a minor effect, larger duration for HFO events across all contacts and MTL and higher frequency of FR across MTL in the non-epileptogenic tissue.

**FIGURE 2:**
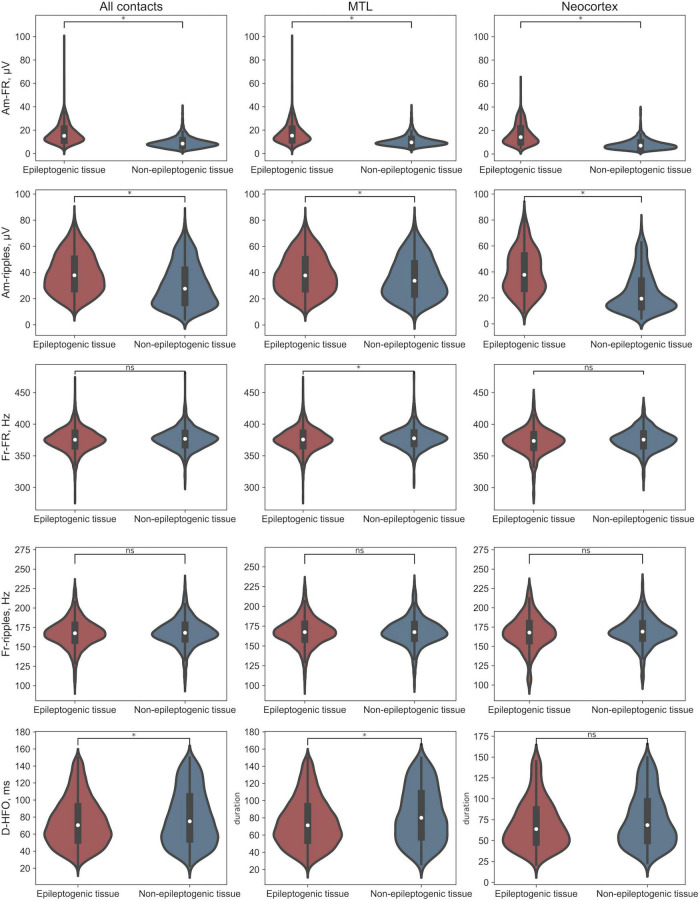
Distributions of HFO morphological features. *Difference significant at α < 0.002 Bonferroni corrected. Am-FR, amplitude of FR; Am-ripples, amplitude of ripples; D-HFO, duration of the co-occurrence of ripple and FR; Fr-FR, frequency of FR; Fr-ripples, frequency of ripples; MTL, mesial temporal lobe.

For the epileptogenic tissue, we observed larger duration for HFO events detected in MTL. Interestingly, no difference was observed in amplitude across MTL and Neocortex in the epileptogenic tissue. In non-epileptogenic tissue, we observed larger amplitude of both FR and ripples, and duration for HFO events detected in MTL. Figure 2 provides features distribution of HFO events across all contacts, separately, in MTL and Neocortex.

#### Cross-validation procedure

**FIGURE 3:**
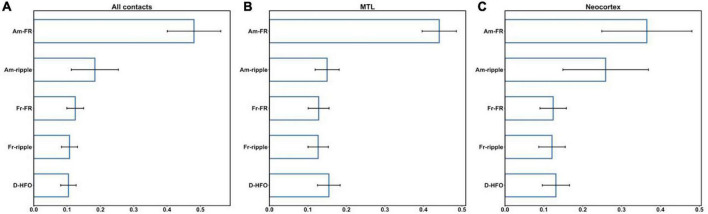
Feature importance across all contacts **(A)**, in MTL **(B),** and Neocortex **(C)**. Am-FR, amplitude of FR; Am-ripples, amplitude of ripples; D-HFO, duration of the co-occurrence of ripple and FR; Fr-FR, frequency of FR; Fr-ripples, frequency of ripples; MTL, mesial temporal lobe.

We evaluated the suitability of HFO morphological features for the classification of HFO events as epileptogenic or not across all contacts and, separately, in MTL and Neocortex (section “Cross-validation procedure”). The CV performed on the RF classifier led to a mean AUC of 83.8% (SD = 1.2%) for all contacts, 79.6% (SD = 0.8%) for MTL, and 86.5% (SD = 0.4%) for the Neocortex. Therefore, HFO morphological features differ between epileptogenic and non-epileptogenic tissues. Figure 3 shows the feature importance provided by the RF classifiers across all contacts, in MTL and Neocortex. For all RF classifiers, the amplitude of FR showed the most discriminant power, followed by the amplitude of ripples in Neocortex. Details on feature importance are reported in the Supplementary material (Supplementary Table 2). The same analysis with leave-one-patient-out CV led to qualitatively similar results (see Supplementary Tables 4, 5).

**FIGURE 4:**
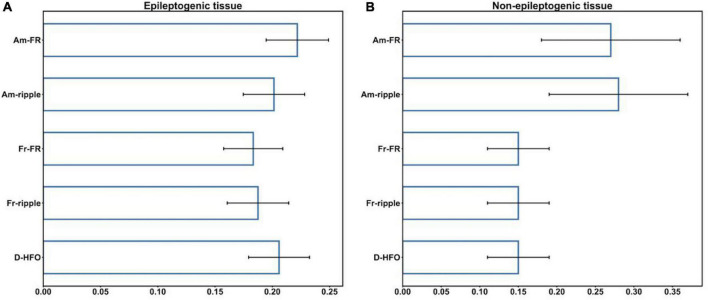
Feature importance inside the epileptogenic **(A)**, non-epileptogenic **(B)** tissues. Am-FR, amplitude of FR; Am-ripples, amplitude of ripples; D-HFO, duration of the co-occurrence of ripple and FR; Fr-FR, frequency of FR; Fr-ripples, frequency of ripples; MTL, mesial temporal lobe.

Additionally, we evaluated the suitability of HFO morphological features for the classification of HFO events between MTL and Neocortex, separately, inside the epileptogenic and non-epileptogenic tissues. The CV performed on the RF classifier led to a mean AUC of 64.2% (SD = 1.0%) for the epileptogenic tissue, 75.7% (SD = 2.3%) for the non-epileptogenic tissue. Thus, epileptogenic HFO have similar morphology in MTL and Neocortex. Figure 4 shows the feature importance of the RF classifiers inside the epileptogenic and non-epileptogenic tissues. While in the epileptogenic tissue all HFO features showed approximately the same discriminant power, in the non-epileptogenic tissue the amplitude of FR and ripples were more prominent. Details on feature importance are reported in the Supplementary material (Supplementary Table 3).

## Discussion

In this study, we prospectively validated HFO rates (the co-occurrence of ripples and FR) as a reliable biomarker for epilepsy surgery outcome. Our analysis followed specific recommendations for a standardized HFO detection pipeline (Fedele et al., 2019) in a newly collected dataset. Moreover, through a machine learning approach, we delineated the distribution of HFO morphological features between epileptogenic and non-epileptogenic tissues across all contacts and, separately, in MTL and Neocortex.

### High accuracy of high-frequency oscillations rates for outcome prediction

High-frequency oscillations rates predicted seizure outcome in the newly collected dataset at the individual patient level with high accuracy. This resulted from the combination of optimal data selection and the usage of a validated HFO detector. Data were selected from long intervals of NREM sleep and provided a stable spatial profile of HFO rates, confirming NREM as an appropriate choice for standardized HFO detection (Nevalainen et al., 2020), even if REM sleep is not yet fully explored (Frauscher and Gotman, 2019). We used an HFO detector adopted in previous studies (Fedele et al., 2017; Boran et al., 2019a,b, 2021; Cserpan et al., 2021; Dimakopoulos et al., 2021), and provided further validation of its usability in the clinical settings with this dataset.

We observed high consistency of HFO analysis with surgical planning in cases with good outcome, which led to high specificity. We correctly predicted good outcomes in 11/14 patients (TN). We had three FP, Patients-8, 11, and 18 (Table 1). In Patient-8 and 11, HFO detected in the precuneus and occipital lobe, respectively, were not resected, while the surgery provided seizure freedom (FP). Importantly, Frauscher et al. (2018) showed during NREM sleep the presence of physiological ripples and FR in the medial parietal lobe due to cognitive processing (Khodagholy et al., 2017; Aleman-Zapata et al., 2020) and, to a large extent, in the occipital lobe. Patient-11 was affected by a periventricular nodular heterotopia (PNH) in the unresected HFO area. Previous studies on HFO in PNH reported high ripple rates (Ferrari-Marinho et al., 2015) not distinguishable from healthy tissue (Pizzo et al., 2017). Therefore, HFO rates alone might not be sufficient for the correct delineation of the epileptogenic tissue in PNH cases. In Patient-18, HFO were detected in both hippocampi, but the resection of the right hippocampus only led to seizure freedom (FP). This is in line with previous studies, that reported the presence of HFO in both hippocampi during the NREM sleep (Staba et al., 2002) and no distinction between the epileptogenic and non-epileptogenic tissues based on HFO rates (Pail et al., 2020).

High PPV mirrors the accurate prediction of poor outcomes in 6/6 patients (TP). Two of them (Patient-1 and 6) were MRI negative with SOZ in the neocortical regions. In both cases, the HFO area pointed to the left hippocampus. HFO in the left hippocampus can reflect the link between epileptogenesis and HFO appearance, which is not necessarily coincident with seizures manifestation (Bragin et al., 2004; Jiruska and Bragin, 2011; Engel and Pitkänen, 2020). Patient-7 had SOZ proximal to gliosis in the lateral temporal lobe (Table 1), while we identified HFO areas in the left hippocampus. This emphasizes that the resection of structural abnormalities does not necessarily lead to good outcomes (Bonilha and Keller, 2015). For Patient-12, the resection of the parietal lobe that did not cover MEC in the temporal lobe caused seizure recurrence. Given that MEC is strongly associated to seizure freedom (Panov et al., 2016), the HFO area found in the MTL near MEC represented further confirmation of epileptogenic tissue. Patient-2 and 17 had SOZ overlapping with the HFO area but extended over eloquent areas. The surgical plan aimed to improve the patient’s condition keeping the eloquent cortex intact.

Interestingly, we did not observe clinical cases with poor outcomes, which were not predicted by the HFO analysis (FN) possibly because of the extensive coverage of the stereo-EEG implantation in our study, which resulted in high sensitivity and NPV. Taken together, the accuracy in outcome prediction based on the HFO rates was 85% in this dataset, which supports the consideration of HFO to improve the quality of surgical planning.

### High-frequency oscillations morphological features

Given our prospective definition of HFO as the co-occurrence of ripples and FR, we detected highly epileptogenic HFO and studied the distribution of HFO morphological features in both ranges separately. Our multivariate approach highlighted that epileptogenic HFO are characterized by larger amplitude in both ripple and FR ranges. Our findings are consistent with previous studies reporting larger amplitude for ripples (von Ellenrieder et al., 2016; Pail et al., 2017; Guragain et al., 2018) and FR (von Ellenrieder et al., 2016; Pail et al., 2017) events in SOZ. Additionally, given the regional specificity of HFO morphology (Frauscher et al., 2018; Weiss et al., 2019), our results showed the delineation between the epileptogenic and non-epileptogenic tissues based on high amplitude ripples more relevant in Neocortex than in MTL. Importantly, we considered here HFO recorded from resected tissue in patients with good outcome, which strengthens our belief that these HFO events delineated the epileptogenic tissue.

Our data did not show prominent differences in the variability of HFO morphology between MTL and Neocortex inside the epileptogenic tissue. This homogeneity of HFO morphological features across anatomical regions supports the generalizability of HFO detection pipelines. Therefore, ripple amplitudes are similar across MTL and Neocortex, but at the same time, ripples are distinct in Neocortex between the epileptogenic and non-epileptogenic tissues. To the best of our knowledge, this is the first report on the characterization of HFO morphology in the epileptogenic tissue, separately, for both ripple and FR ranges. When looking at physiological HFO detected outside of the resected areas, we observed a larger amplitude in both ripple and FR ranges in MTL than Neocortex. This corroborates previous findings (Guragain et al., 2018), providing evidence for a common trend in both ripple and FR ranges.

In a broader view, the implementation of machine learning approaches in clinical neurophysiology is constantly increasing (Cimbalnik et al., 2018, 2019; Weiss et al., 2019; Segato et al., 2020). In this context, mapping HFO morphological features might potentially contribute toward the usage of HFO in the clinical settings (Jacobs and Zijlmans, 2020). In particular, the application of machine learning in multicenter studies might benefit the prognostic value of HFO in the development of epilepsy (Reid et al., 2016), the reliability of HFO to predict the occurrence of seizures (Malinowska et al., 2015), and to mirror disease severity (Boran et al., 2019a).

### Limitations

We acknowledge some limitations in our study. First, the cohort included a limited number of patients, which restricted the variety of the clinical cases in our study. This is partially compensated for by the extensive anatomical coverage provided by the stereo-EEG, which did not affect the specificity of our approach. Importantly, a high number of electrode contacts in the individual patient ensures a more stable estimation of the HFO rate threshold needed to delineate the HFO area. We believe that our pipeline needs further validation in a larger cohort based on the promising results obtained on this dense spatial sampling. Second, our study design dichotomizes between cases with fully resected and partially or unresected epileptogenic tissues as good and poor outcomes. Therefore, we are not considering the post-surgical improvement in ILAE = 2–4 poor outcomes, even when HFO were partially resected. Third, while all HFO were detected with the same criteria, we cannot assume that all HFO outside the resection were epileptogenic, as this could be unequivocally proved only by a second resection. Fourth, while we established an association between the HFO area and the epileptogenic tissue, the extension of the latter remains unknown. However, the location of the HFO area remains a reliable indicator of which contacts demonstrate interictal epileptogenic activity. Fifth, HFO morphological features might differ among anatomical regions inside MTL and Neocortex. A larger dataset, possibly obtained by a multicenter study, might permit a more detailed anatomical mapping of HFO morphological features.

## Conclusion

In conclusion, we prospectively validated the predictive value of HFO rates against seizure outcome. Following previous recommendations on HFO analysis (Fedele et al., 2019), we obtained an accuracy of 85% in a newly collected dataset. Next, we showed that HFO morphological features in HFO events predictive of good outcome are stable across brain regions, while HFO amplitude tends to be larger in the epileptogenic tissue in both MTL and Neocortex. We believe that a multicenter study with uniform and standardized data processing might support the generalization of our findings.

## Data availability statement

The original contributions presented in this study are included in the article/Supplementary material, further inquiries can be directed to the corresponding author.

## Ethics statement

The studies involving human participants were reviewed and approved by the National Medical and Surgical Center named after N.I. Pirogov Ethics Committee. The patients/participants provided their written informed consent to participate in this study.

## Author contributions

VK: conceptualization, methodology, investigation, data curation, formal analysis, software, writing – original draft, and project administration. AB and NU: methodology, data curation, and validation. NP and AZ: methodology, data curation, and resources. OD: methodology, data curation, resources, and funding acquisition. TF: conceptualization, methodology, investigation, formal analysis, software, writing – review and editing, supervision, and funding acquisition. All authors contributed to the article and approved the submitted version.

## Footnotes

1
https://github.com/vkarpychev/Morphological-features-of-HFO
