# Supplementary material for: Epileptogenic high-frequency oscillations present larger amplitude both in mesial temporal and neocortical regions
Source: Front Hum Neurosci. 2022 Sep 29;16:984306. doi: 10.3389/fnhum.2022.984306 (PMC9557004; doi:10.3389/fnhum.2022.984306)
Supplement: Supplementary file 1 [file Data_Sheet_1.PDF]

Supplementary material

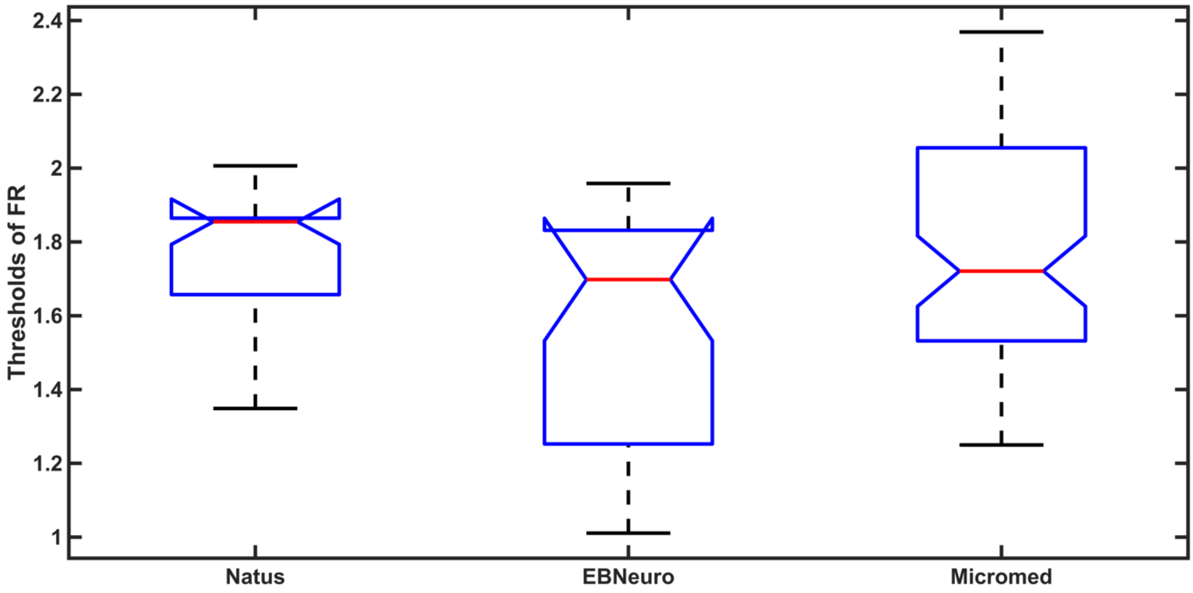

Figure S1. *Noise levels in the fast ripples range across Natus, EBNeuro, and Micromed acquisition systems.* We considered the amplitude threshold computed for the detection of FR (250-500 Hz) representative of the background noise level. For each patient we computed the noise level with low HFO rate for all available 5 minutes intervals. Kruskal-Wallis test indicated no significant difference ($H(2) = 4.7$, $p = .09$) across Natus (mean = 1.78, $SD = 0.17$), EBNeuro (mean = 1.58, $SD = 0.34$), and Micromed (mean = 1.77, $SD = 0.29$) acquisition systems.

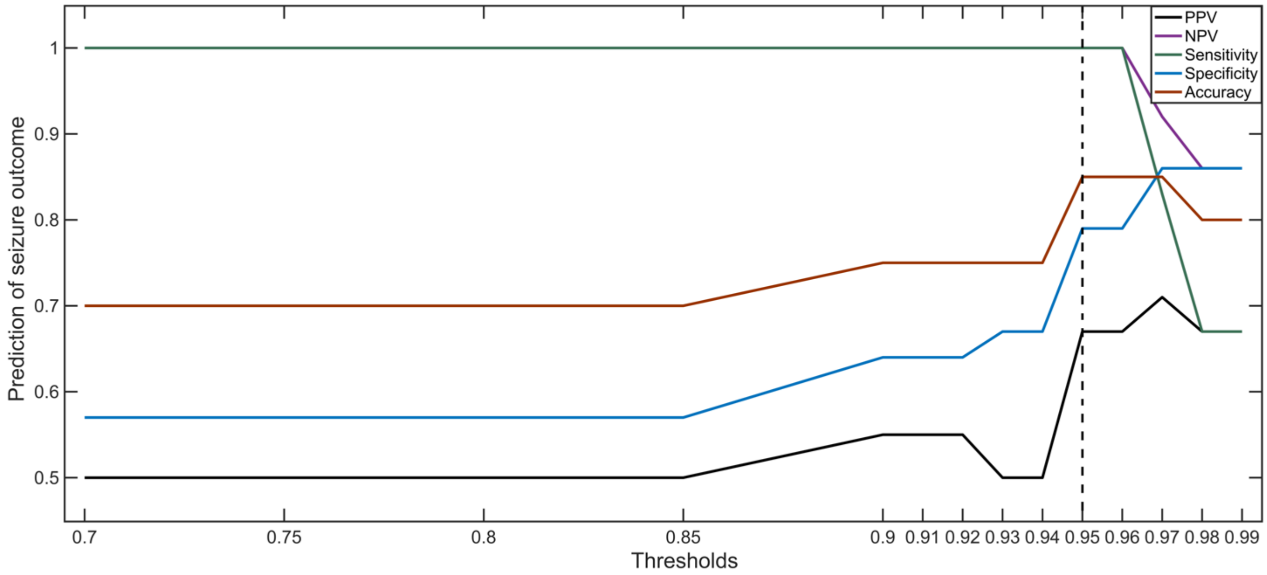

Figure S2. *Prediction of seizure outcome across different thresholds of the HFO rate distribution.* The positive predictive value (PPV) is highlighted in black; the negative predictive value (NPV) is highlighted in purple; sensitivity is highlighted in green; specificity is highlighted in blue; accuracy is highlighted in red. The 95%-threshold is shown as a black dotted line.

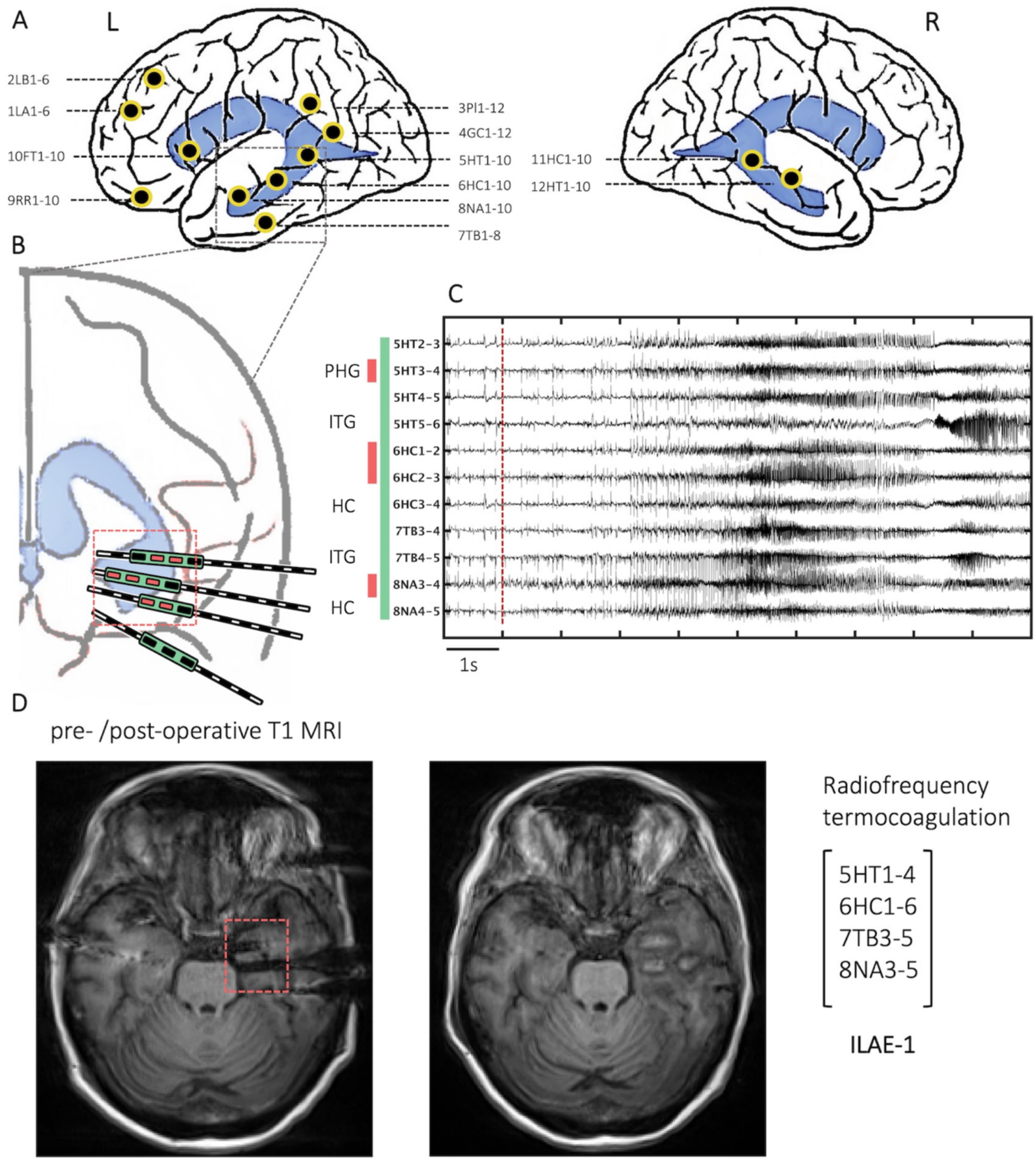

Figure S3. *The analysis of Patient-16 (good outcome - TN).* **(A)** Stereo-EEG implantation scheme. The stereo-EEG implantation scheme was adopted from Mullin, J. P., Smithason, S., & Gonzalez-Martinez, J. (2016). Stereo-Electro-Encephalo-Graphy (SEEG) With Robotic Assistance in the Presurgical Evaluation of Medical Refractory Epilepsy: A Technical Note. *J Vis Exp: JoVE*, (112), 53206. https://doi.org/10.3791/53206. **(B)** The channels representing the SOZ are marked in green, HFO area is marked in red. HFO area is additionally highlighted with the red dotted box. The SOZ and HFO area were found in the left mesial temporal lobe. **(C)** The stereo-EEG signals at the seizure onset, highlighted with the red dotted line. **(D)** Pre-operative T1 MRI (left) and post-operative T1 MRI (right). The red dotted box highlighted the HFO area. The HFO area was included in the resection area (left mesial temporal lobe), which led to good outcome (TN).

ILAE = International League Against Epilepsy; ITG = inferior temporal lobe; HC = hippocampus; L = left; PHG = parahippocampal gyrus; R = right.

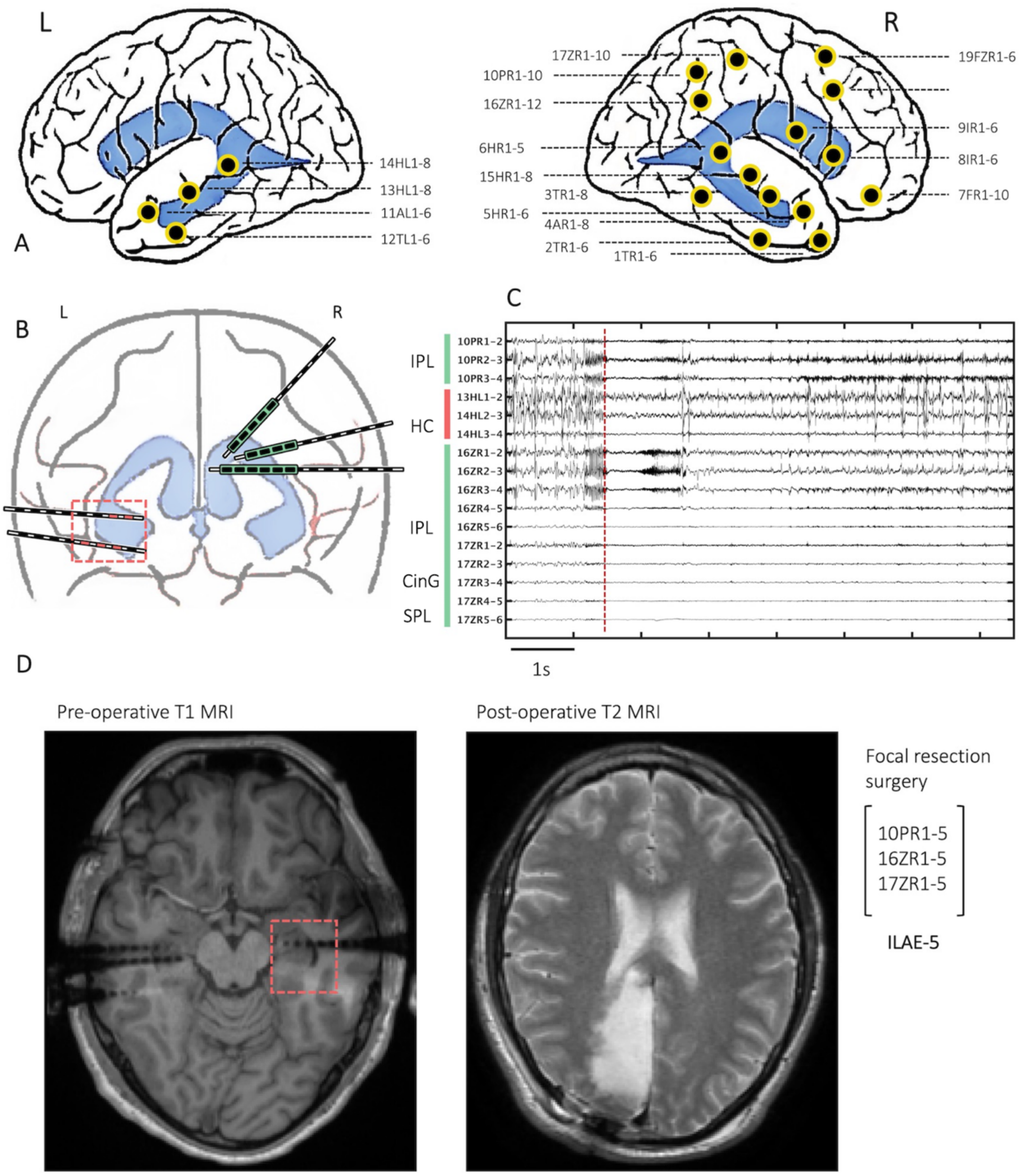

Figure S4. *The analysis of Patient-12 (poor outcome - TP).* **(A)** Stereo-EEG implantation scheme. The stereo-EEG implantation scheme was adopted from Mullin, J. P., Smithason, S., & Gonzalez-Martinez, J. (2016). Stereo-Electro-Encephalo-Graphy (SEEG) With Robotic Assistance in the Presurgical Evaluation of Medical Refractory Epilepsy: A Technical Note. *J Vis Exp: JoVE*, (112), 53206. https://doi.org/10.3791/53206. **(B)** The channels representing the SOZ are marked in green, HFO area is marked in red. HFO area is additionally highlighted with the red dotted box. The SOZ was found in the right parietal lobe and the HFO area in the left mesial temporal lobe. **(C)** The stereo-EEG signals at the seizure onset, highlighted with the red dotted line. **(D)** Pre-operative T1 MRI (left) and post-operative T2 MRI (right). The red dotted box highlighted the HFO area. The HFO area (left mesial temporal lobe) was not included in the resection area (right parietal lobe), which led to poor outcome (TP).

ILAE = International League Against Epilepsy; CinG = cingulate gyrus; IPL = inferior parietal lobule; HC = hippocampus; L = left; R = right; SPL = superior parietal lobule.

Table S1.

Overlapping between spikes and HFO events

ID	Total number of spikes	Spike peak overlapping with HFO [window of 200 ms],%	HFO containing the spike peak [window of 200 ms],%
1	55	62.0 [100.0]	17.0 [24.0]
2	33	30.0 [82.0]	4.0 [10.0]
3	20	80.0 [95 .0]	7.0 [8.0]
4	4	75.0 [100.0]	1.0 [2.0]
5	2	100.0 [100.0]	1.0 [1.0]
6	43	40.0 [58.0]	13.0 [19.0]
7	18	28.0 [44.0]	1.0 [1.0]
8	4	75.0 [100.0]	1.0 [1.0]
9	6	83.0 [83.0]	4.0 [4.0]
10	5	100.0 [100.0]	3.0 [4.0]
11	17	59.0 [76.0]	4.0 [5.0]
12	44	18.0 [57.0]	2.0 [6.0]
13	52	62.0 [100.0]	14.0 [24.0]
14	45	49.0 [64.0]	10.0 [13.0]
15	71	32.0 [49.0]	5.0 [8.0]
16	151	44.0 [54.0]	22.0 [27.0]
17	30	57.0 [80.0]	15.0 [21.0]
18	1	0.0 [0.0]	0.0 [0.0]
19	59	76.0 [86.0]	30.0 [34.0]
20	4	50.0 [50.0]	1.0 [1.0]

Note. For each patient, we visually marked spike peaks in the channel with the highest HFO rates during one 5-minute NREM interval. We considered the overlapping of the spike peaks with the HFO events and the overlap of a temporal window of 200 ms around the spike peaks (reported inside brackets). We observed 56.0% (*SD* = 26.0%) of spike peaks overlapping with HFO and 8.0% (*SD* = 8.0%) of HFO overlapping with spike peaks. Given a window of 200 ms around each spike peak, we observed 73.0% (*SD* = 26.0%) of spikes overlapping with HFO and 11.0% (*SD* = 10.0%) of HFO overlapping with spikes. Thus, HFO and spikes provide different information.

Table S2.

Feature importance of RF classifiers between Class-1 and Class-2 across all contacts, in MTL and Neocortex

	**All contacts**		**MTL**		**Neocortex**	
	M	*SD*	*M*	*SD*	*M*	*SD*
Am-FR	0.48	0.08	0.44	0.05	0.37	0.12
Am-ripples	0.18	0.07	0.15	0.03	0.26	0.11
Fr-FR	0.11	0.02	0.13	0.03	0.12	0.03
Fr-ripples	0.10	0.02	0.13	0.03	0.12	0.03
D-HFO	0.12	0.03	0.15	0.03	0.13	0.04

Note. Am-FR = amplitude of FR; Am-ripples = amplitude of ripples; D-HFO = duration of the co-occurrence of ripple and FR; Fr-FR = frequency of FR; Fr-ripples = frequency of ripples; MTL = mesial temporal lobe; M = mean; SD = standard deviation.

Table S3.

Feature importance of RF classifiers between MTL and Neocortex inside Class-1 and Class-2

	**Class-1**		**Class-2**	
	M	*SD*	*M*	*SD*
Am-FR	0.22	0.03	0.27	0.09
Am-ripples	0.20	0.03	0.28	0.09
Fr-FR	0.18	0.03	0.15	0.04
Fr-ripples	0.19	0.03	0.15	0.04
D-HFO	0.21	0.03	0.15	0.04

Note. Am-FR = amplitude of FR; Am-ripples = amplitude of ripples; D-HFO = duration of the co-occurrence of ripple and FR; Fr-FR = frequency of FR; Fr-ripples = frequency of ripples; MTL = mesial temporal lobe; M = mean; SD = standard deviation.

Table S4.

Comparison of AUC scores between 5-fold CV and leave-one-out-patient CV

	All contacts	MTL	Neocortex
	M [*SD*], %	*M* [*SD*], %	*M* [*SD*], %
5-fold CV	83.8 [1.2]	79.6 [0.8]	86.5 [0.4]
Leave-one-patient-out CV	73.0 [6.0]	68.0 [11.0]	65.0 [7.0]

Note. We evaluated the RF classifier through the leave-one-patient-out CV, as well. For each iteration, the data of one patient was used as a testing set, whereas the RF classifier trained on the data of the rest patients. Like with the 5-fold CV (*Section 2.7.2*), we applied *a Grid search* algorithm to find the optimal hyperparameters in each iteration and balanced the number of HFO events during the training by the *SMOTE* approach using the package *'imbalanced-learn'*. We quantified the performance of each RF classifier computing the area under the receiver operating characteristic curve (AUC) and the importance of each feature using the mean decrease in the *Gini index*. The leave-one-patient-out CV provided lower performance and high variance.

CV = cross-validation; MTL = mesial temporal lobe; M = mean; SD = standard deviation.

Table S5.

Comparison of feature importance between 5-fold CV and leave-one-out-patient CV

	All contacts		MTL		Neocortex	
	5-fold CV	Leave-one-patient-out CV	5-fold CV	Leave-one-patient-out CV	5-fold CV	Leave-one-patient-out CV
	M [*SD*]	*M* [*SD*]	*M* [*SD*]	*M* [*SD*]	*M* [*SD*]	*M* [*SD*]
Am-FR	0.48 [0.08]	0.38 [0.02]	0.44 [0.05]	0.37 [0.02]	0.37	0.22 [0.01]
Am-ripples	0.18 [0.07]	0.20 [0.01]	0.15 [0.03]	0.17 [0.01]	0.26	0.21 [0.01]
Fr-FR	0.11 [0.02]	0.13 [0.01]	0.13 [0.03]	0.14 [0.01]	0.12	0.18 [0.01]
Fr-ripples	0.10 [0.02]	0.14 [0.01]	0.13 [0.03]	0.14 [0.01]	0.12	0.18 [0.01]
D-HFO	0.12 [0.03]	0.15 [0.01]	0.15 [0.03]	0.17 [0.01]	0.13	0.19 [0.01]

Note. For the leave-one-patient-out CV, the feature importance resembles the results obtained for the 5-fold CV.

Am-FR = amplitude of FR; Am-ripples = amplitude of ripples; D-HFO = duration of the co-occurrence of ripple and FR; Fr-FR = frequency of FR; Fr-ripples = frequency of ripples; MTL = mesial temporal lobe; M = mean; SD = standard deviation.
